# Evaluation of the immunogenicity and protective efficacy of an inactivated vaccine candidate for sheep infected with ovine parainfluenza virus type 3

**DOI:** 10.1186/s13567-024-01339-1

**Published:** 2024-06-27

**Authors:** Yanhua Ma, Jialei Wang, Youzhi Wu, Xiaohui Zan, Yan Wang, Yanyan Zhou, Tao Wang, Caifeng Gong, Kai Meng, Rui Niu, Qiang Shang, Hao Wang, Jiali Wang, Ying He, Wei Wang

**Affiliations:** 1 State Key Laboratory of Reproductive Regulation and Breeding of Grassland Livestock, School of Life Sciences, Inner Mongolia University, Hohhot, China; 2 Basic Medical School, Inner Mongolia Medical University, Hohhot, China; 3 Inner Mongolia Mengwei Biotech Co. Ltd, Hohhot, 012000 China; 4 Animal Epidemic Prevention Service Center of Jining, Ulanqab, China

**Keywords:** Ovine parainfluenza virus type 3, inactivated OPIV3 vaccine, pathogenicity, respiratory disease

## Abstract

Respiratory diseases constitute a major health problem for ruminants, resulting in considerable economic losses throughout the world. Parainfluenza type 3 virus (PIV3) is one of the most important respiratory pathogens of ruminants. The pathogenicity and phylogenetic analyses of PIV3 virus have been reported in sheep and goats. However, there are no recent studies of the vaccination of sheep or goats against PIV3. Here, we developed a purified inactivated ovine parainfluenza virus type 3 (OPIV3) vaccine candidate. In addition, we immunized sheep with the inactivated OPIV3 vaccine and evaluated the immune response and pathological outcomes associated with OPIV3 TX01 infection. The vaccinated sheep demonstrated no obvious symptoms of respiratory tract infection, and there were no gross lesions or pathological changes in the lungs. The average body weight gain significantly differed between the vaccinated group and the control group (*P* < 0.01). The serum neutralization antibody levels rapidly increased in sheep post-vaccination and post-challenge with OPIV3. Furthermore, viral shedding in nasal swabs and viral loads in the lungs were reduced. The results of this study suggest that vaccination with this candidate vaccine induces the production of neutralizing antibodies and provides significant protection against OPIV3 infection. These results may be helpful for further studies on prevention and control strategies for OPIV3 infections.

## Introduction

Parainfluenza virus type 3 (PIV3) is one of the most important viral respiratory pathogens for humans and many species of animals [1]. The virus belongs to the family Paramyxoviridae, subfamily Orthoparamyxovirinae, genus Respirovirus, which includes human parainfluenza virus type 3 (HPIV3), bovine parainfluenza virus type 3 (BPIV3), caprine parainfluenza virus type 3 (CPIV3), human parainfluenza virus type 1 (HPIV1), murine parainfluenza virus 1 (MPIV1) and other newly discovered strains. The members of this virus family are enveloped and have genomes consisting of a single segment of negative-sense RNA, which usually has a length of 15 kb [2, 3]. PIV3 infections are found in a wide variety of mammals, including humans, cattle, sheep, goats, rhinoceros, pigs, dogs, dolphins, bison, guinea pigs, moose, bighorn sheep, camels, and water buffaloes [1, 4–7].

PIV3 infection usually results in respiratory symptoms and increases morbidity and mortality rates under a variety of stress conditions or during coinfection with other bacterial pathogens [7–9]. Immunization is the most effective method for preventing infectious diseases. Therefore, the rapid development of effective vaccines against PIV3 is urgently needed.

HPIV3 is one of the most common viral pathogens that causes acute lower respiratory infection (ALRI) in children, particularly in infants. Currently, no vaccine exists for the virus [10]. Over the last decade, several HPIV3 live-attenuated vaccines, such as rHPIV3cp45, rB/HPIV3, and MEDI-534, have been tested in clinical trials [11]. In addition, several vaccines are being developed to prevent HPIV3 infection, including those containing viral epitopes, DNA- and RNA-based formulations, adenovirus-based vectors, and inactivated whole viruses [12–15].

BPIV3 infection causes severe bronchopneumonia and secondary bacterial infections in instances of high stress, such as during transportation, weather change and feedlot conditions [16]. The main clinical signs of BPIV3 infection are coughing, anorexia, pyrexia, nasal and ocular discharge, dyspnoea and sometimes diarrhoea [17]. Shortly after the discovery of BPIV3, the first inactivated vaccines against this virus were developed [18], followed by modified live virus (MLV) vaccines [19]. BPIV3 is closely related to bovine respiratory syncytial virus (BRSV), bovine infectious rhinotracheitis virus (IBR), bovine viral abdomen diarrhoea-mucocoosis virus (BVDV), and bovine adenovirus (BADV), and mixed infections with these viruses can occur, exacerbating their effect on the immune system [20–22]. Thus, several studies have been performed to investigate the possibility of combining BRSV-BPIV3 vaccines with other vaccines, for example, the combination of a live BRSV-BPIV3 vaccine with an *M. haemolytica* vaccine or the combination of an inactivated BRSV-BPIV3-*M. haemolytica* vaccine with a live bovine herpesvirus vaccine [23, 24]. In particular, BPIV3 is a promising vaccine vector for the treatment of infections caused by various respiratory viruses, including HPIV3, respiratory syncytial virus (RSV), and severe acute respiratory syndrome-coronavirus 2 (SARS-CoV-2) [25].

Currently, there are only two complete gene sequences of sheep-sourced parainfluenza viruses in the GenBank database, namely, TX01 (GenBank No. MT756864) and TJ2022 (GenBank No. OR472985.1), while the others are goat-sourced parainfluenza viruses. Compared to HPIV3 and BPIV3, there are fewer sheep-sourced parainfluenza virus strains, and unlike BPIV3, they have not been typed. Although the isolation of parainfluenza virus type 3 from sheep was reported in 1966 [26], epidemiological surveys have shown that PIV3 remains widely prevalent in sheep and goat flocks [7, 27, 28]. Similar to BPIV3, PIV3 infection usually results in respiratory symptoms in goats and sheep. The noted clinical signs include coughing, nasal discharge, dyspnoea and anorexia. Gross and histopathological lesions are mainly found in the lungs and trachea [29, 30]. However, there are no commercial PIV3 vaccines available for sheep and goats or prevention and control measures available. In this study, an OPIV3 challenge model was established in lambs. An inactivated ISA 206 adjuvant OPIV3 vaccine candidate was also developed. Then, the immunogenicity and protective efficacy of the PIV3 vaccine were evaluated in sheep challenged with OPIV3.

Two-month-old sheep subjected to PIV3 inactivation showed a significantly lower temperature response, gross pathology and PIV3 replication in the lungs than did the control group. These results indicate that sheep immunization with an inactivated vaccine candidate for OPIV3 confers protection against PIV3, supporting this strategy for respiratory diseases.

## Materials and methods

### Cell lines and virus culture

The viral stock was propagated in Madin–Darby bovine kidney (MDBK) cells cultured with Dulbecco’s modified Eagle’s medium (DMEM) (GIBCO, USA) supplemented with 1% penicillin/streptomycin and 2% foetal bovine serum (FBS) and incubated at 37 °C in a 5% CO_2_ incubator. When the cytopathic effect (CPE) exceeded 80%, the cell culture supernatant was collected, purified by low-speed centrifugation (300 × *g* for 10 min), and stored at −80 °C.

### Viral titration

Viral titration was determined by a 50% cell culture infectious dose (TCID_50_) endpoint dilution assay. Briefly, MDBK cells were inoculated in a 96-well culture plate and cultured until they reached 70–80% confluence. Then, the supernatant was removed, and the cells were washed twice with phosphate-buffered saline (PBS). Serial tenfold dilutions of the viruses were added to the samples, which were subsequently plated in a 96-well culture plate. After 5 days of culture at 37 °C, the cells were checked under a microscope for the presence of CPE.

### TEM observation

The virus was stained with 2% phosphotungstate acid for 1–2 min at room temperature and examined via transmission electron microscopy (TEM, JEM-1400 FLASH, Japan Electronics).

### Preparation of the inactivated OPIV3 vaccine

The OPIV3 viruses cultured for the inactivated vaccine were generated based on previous methods.

To prepare the virus-inactivated solution, 0.2 mol/L BEA was mixed with 0.4 mol/L NaOH at a volume ratio of 2:1 in the appropriate container at 37 °C for at least 1 h to generate the BEI solution, after which the BEI solution was filtered through a 0.22 μm membrane (Millipore). To hydrolyse residual BEI, 2% sodium thiosulfate was added to the filtered BEI solution at a final concentration of 0.03 mol/L in a warm bath at 37 °C for 1 h with gentle stirring for 10 min. The conditions for OPIV3 viral inactivation were determined with a set volume and concentration of the BEI solution in this study. To validate effective virus inactivation, 0.1 mL of inactivated virus was inoculated from each of the virus/BEI mixtures into MDBK cell culture flasks at 37 °C for 5 days. Then, the flasks were freeze-thawed three times, and 0.1 mL of purified supernatant was transferred to another MDBK cell monolayer in a 25 cm^2^ flask, which was incubated at 37 °C for another 5 days. The passage process was repeated three times. No CPE was observed after three passages, while positive controls showed 100% CPE.

For the OPIV3 vaccine formulation, the ISA 206 adjuvant (SEPPIC, France) was used. The ISA 206 adjuvant was autoclaved at 121 °C for at least 40 min. Before emulsification, the liquid temperature was controlled within the range of 28–32 °C. The inactivated antigen was mixed with the ISA 206 adjuvant at a ratio of 50% by weight. For a stable W/O/W vaccine emulsion, a one-step process was performed using a low shear rate and controlled temperature at 31 °C (± 1 °C) for 25 min until the antigen was fully mixed with the adjuvant. The duplex oil emulsion was kept at 4 °C until use.

### Sheep immunization, infection, and sample collection

Fifteen 2-month-old sheep (Du Han hybrid sheep) were obtained from a farm in Inner Mongolia, China. All animals were negative for OPIV3, *Mycoplasma ovipneumoniae* (MO), *Mannheimia haemolytica* (M.H.), and brucellosis and presented no signs of depression, cough or other health disorders. The animals were randomly divided into three groups of five sheep each, including a vaccinated group, a control group and a nonchallenged group.

The vaccinated group was administered 2 mL of inactivated vaccine per sheep via intramuscular (IM) injection. After 21 days of vaccination, the sheep were boosted via the same dose and delivery route as above. After 42 days, the vaccinated group and control group were challenged with 4 mL of OPIV3 at 1 × 10^7.5^ TCID_50_/mL via tracheal injection. The sheep in the nonchallenged group remained unvaccinated and unchallenged and served as the negative control group. The rectal temperature and body weights were monitored in the animal experiment. Blood samples were collected from the sheep before immunization and every 7 days after vaccination and booster immunization. The blood was centrifuged, and the serum was collected and stored at −20 °C for antibody detection. Clinical signs, including depression, cough, asthma, and other respiratory symptoms, were recorded from Day 0 to Day 14, and “+” indicates that one of the above clinical symptoms occurred. In addition, the number of “+” represents the severity,+mild;+ +, moderate;+++ , severe. The data were scored on a scale of 0–3 (0 = absent; 1 = mild; 2 = moderate; 3 = severe). On the 14th day after infection, all experimental animals were humanely euthanized. Nasal swab specimens and lungs were collected. All of the respective animal protocols were reviewed and approved by the Institutional Animal Ethics Committee of Inner Mongolia University (Approval No. IMU-2021-sheep-044).

### Gross pathology and histopathology

On Day 14, gross dissection was performed. The gross lesions in the lungs, tracheas, lymph nodes and other tissues of the sheep were observed and examined via necropsy. The extent of gross lesions was evaluated and is expressed as normal, mild, moderate or severe. Liver, spleen, lung, heart, kidney, intestine, and mesenteric lymph node tissue samples were collected and fixed in buffer with 10% formalin for histopathological analysis.

### Immunohistochemistry (IHC)

Formalin-fixed lungs were subjected to immunohistochemistry (IHC) to evaluate the OPIV3 load. The sections were incubated with 500-fold dilutions of monoclonal antibodies against the N protein of OPIV3 (developed in our laboratory) at 4 °C overnight, followed by incubation with 100-fold diluted goat anti-mouse IgG-HRP (Abcam 205719) at 37 °C for 50 min. Then, freshly prepared DAB (Servicebio G1211, Wuhan, China) was added to the sections for colour development at room temperature for approximately 10 min. Finally, the sections were stained with Mayer’s haematoxylin (Servicebio G1004, Wuhan, China) for 1 min, dehydrated and mounted with neutral gum. The percentage of immunostaining and the staining intensity (0, negative; 1+ , weak; 2+ , moderate; and 3+ , strong) were recorded (ImageScope, Leica). The H-score was calculated using the following formula:

H-SCORE = ∑(PI × I) = (percentage of cells with weak intensity × 1) + (percentage of cells with moderate intensity × 2) + percentage of cells with strong intensity × 3).

### Real-time quantitative RT-PCR (qRT-PCR)

Total RNA was extracted from 100 mg of lung homogenate with TRIzol reagent (Takara, China) according to the manufacturer’s instructions. cDNA was synthesized using a Takara 6210A PrimeScript™II 1st Strand cDNA Synthesis Kit. qPCR was performed using TB Green Premix Ex Taq II (Takara, China) according to the manufacturer’s instructions. OPIV3-specific primers (forward primer 5′-AGGTAGGCAATCCACCAAAGC-3′, reverse primer 5′-CCCGATTGGTAAAGAACCTGAT-3′) were used for amplification. The samples were heated to 95 °C for 30 s, followed by 45 cycles of 10 s at 95 °C and 30 s at 60 °C. The negative and positive controls contained ddH_2_O, and the standard plasmid was included in the experiments. The viral genome copy number was calculated according to the results from the tenfold serial dilutions of the positive plasmid. All of the reactions were performed in triplicate.

### Virus neutralization assay

Serum samples collected from immunized animals were inactivated at 56 °C for 0.5 h and serially diluted with cell culture medium in two steps. The diluted sera were mixed with a virus suspension of 100 TCID_50_ in 96-well plates at a ratio of 1:1, followed by 2 h of incubation at 37 °C in a 5% CO_2_ incubator. The serum-virus mixture was subsequently added to a 96-well culture plate supplemented with 1 × 10^4^ MDBK cells, after which the plates were incubated for 5 days at 37 °C in a 5% CO_2_ incubator. The CPE of each well was recorded under a microscope, and the neutralizing titre was calculated by diluting the sample to 50%.

### Statistical analysis

Statistical analyses were performed using Graph Pad Prism 6.02 software.

## Results

### Characterization of the OPIV3 vaccine candidate

**Figure 1:**
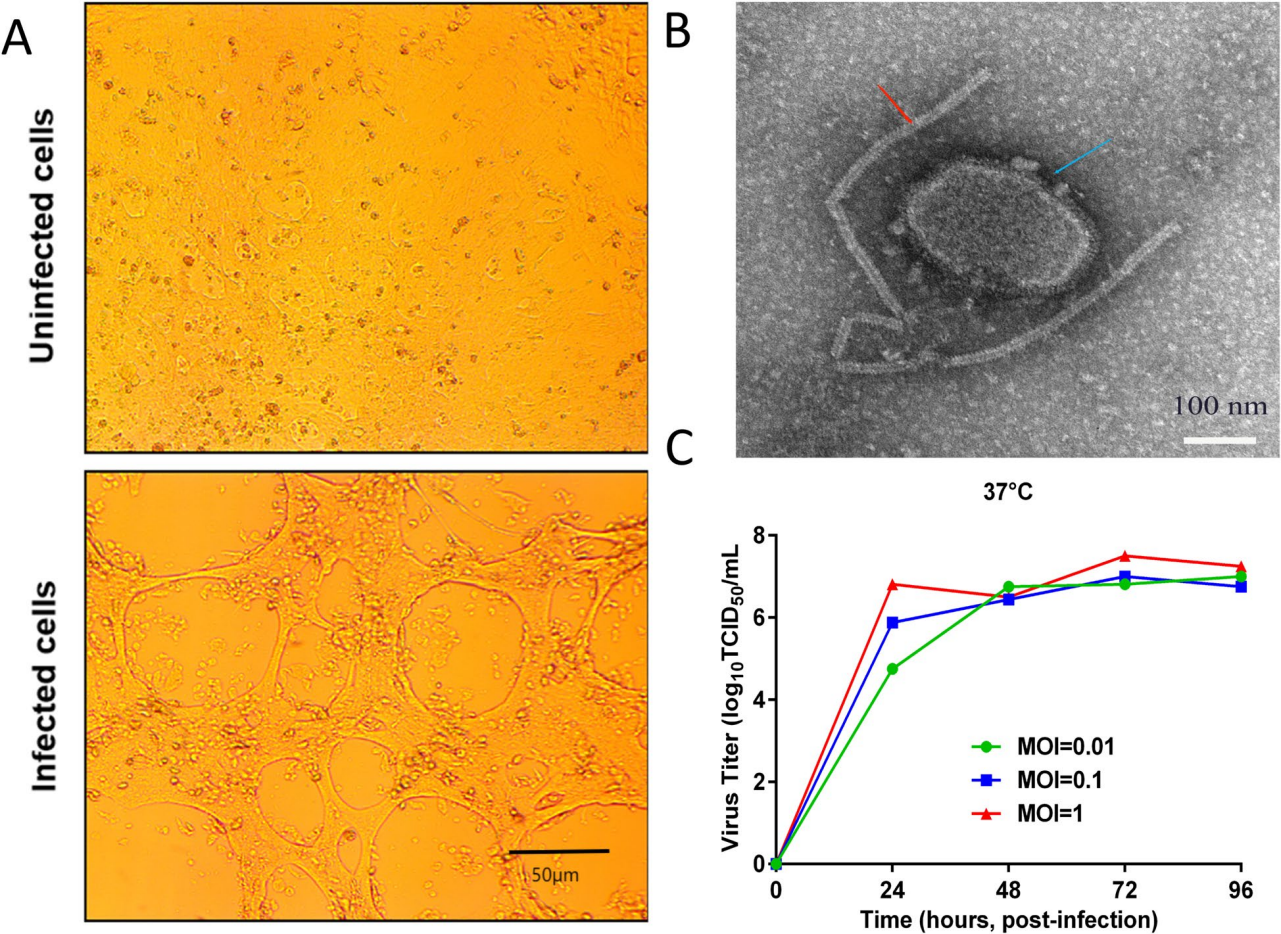
**Characterization of the OPIV3 vaccine candidate.** **A** Confirmation of OPIV3 infection in MDBK cells. OPIV3-induced CPE was observed, and the cell monolayer was completely destroyed after OPIV3 infection (scale bar = 50 nm). **B** TEM image of OPIV3 (scale bar = 100 nm). A micrograph of OPIV3 showed intact, oval-shaped particles with diameters ranging from 150 to 250 nm (shown by the blue arrow). A long fragment of free nucleocapsid chains originated from the disintegration of viral particles in the supernatant of a CPE-positive MDBK cell culture infected with the OPIV3 TX01 strain (shown by the red arrow). **C** Growth kinetics of MDBK cells infected with OPIV3 at 0.01, 0.1 and 1.0 MOI.

To obtain a viral stock that efficiently replicates in MDBK cells for OPIV3 vaccine production, the OPIV3 TX01 strain was passaged in MDBK cells to generate the P21 stock virus. MDBK cells infected with the OPIV3 TX01 strain exhibited CPE at 72 h post-infection (Figure 1A). Transmission electron microscopy was used to observe OPIV3. The micrograph of OPIV3 showed intact, oval-shaped particles with diameters of 150 to 250 nm (blue arrow). A long fragment of free nucleocapsid chains originated from the disintegration of the viral particles in the supernatant of a CPE-positive MDBK cell culture infected with the OPIV3 TX01 strain (red arrow) (Figure 1B). The OPIV3 TX01 strain replicated efficiently and reached a peak titre at 10^7.5^ TCID_50_/mL after 72 h at an MOI of 1.0 (Figure 1C).

### OPIV3 vaccine preparation

**Figure 2:**
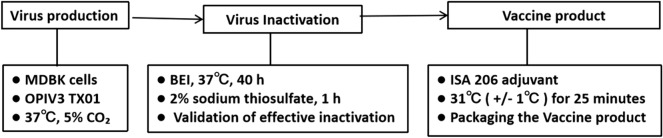
**Flowchart of OPIV3 vaccine preparation.**

The OPIV3 virus was cultured in 175 cm^2^ flasks and inactivated with 0.004 mol/L BEI for 40 h at 37 °C under gentle rocking for viral inactivation. The inactivated virus/BEI solution was neutralized by the addition of 2% sodium thiosulfate. The purified viruses were formulated with ISA 206 adjuvant as the OPIV3 vaccine. The working flowchart of OPIV3 vaccine preparation is shown in Figure 2.

### Vaccination protects sheep against challenge with the OPIV3 TX01 F6 strain

**Figure 3:**
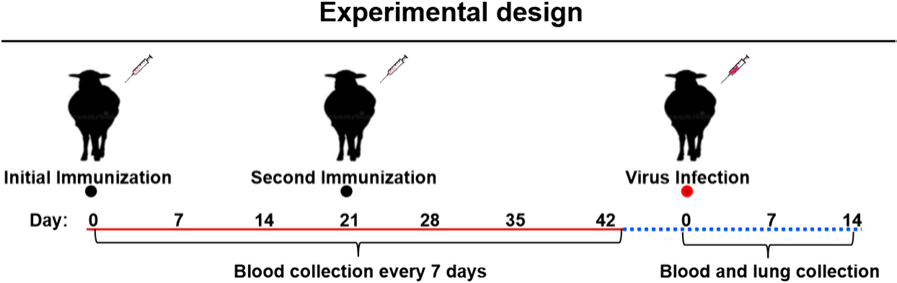
**Animal experimental design.** Sheep were intramuscularly immunized with inactivated OPIV3 TX01 at Days 0 and 21 and challenged with 4 mL of OPIV3 (10^7.5^ TCID_50_/mL) after Day 42.

A flowchart of the vaccine immunogenicity analysis is shown in Figure 3. After vaccination with the inactivated OPIV3 vaccine candidate, all of the animals except one showed an increase in body temperature of approximately 40.0 °C for a single day from 0 to 5 days post-vaccination. Adverse effects were not observed in any of the vaccinated animals.

**Figure 4:**
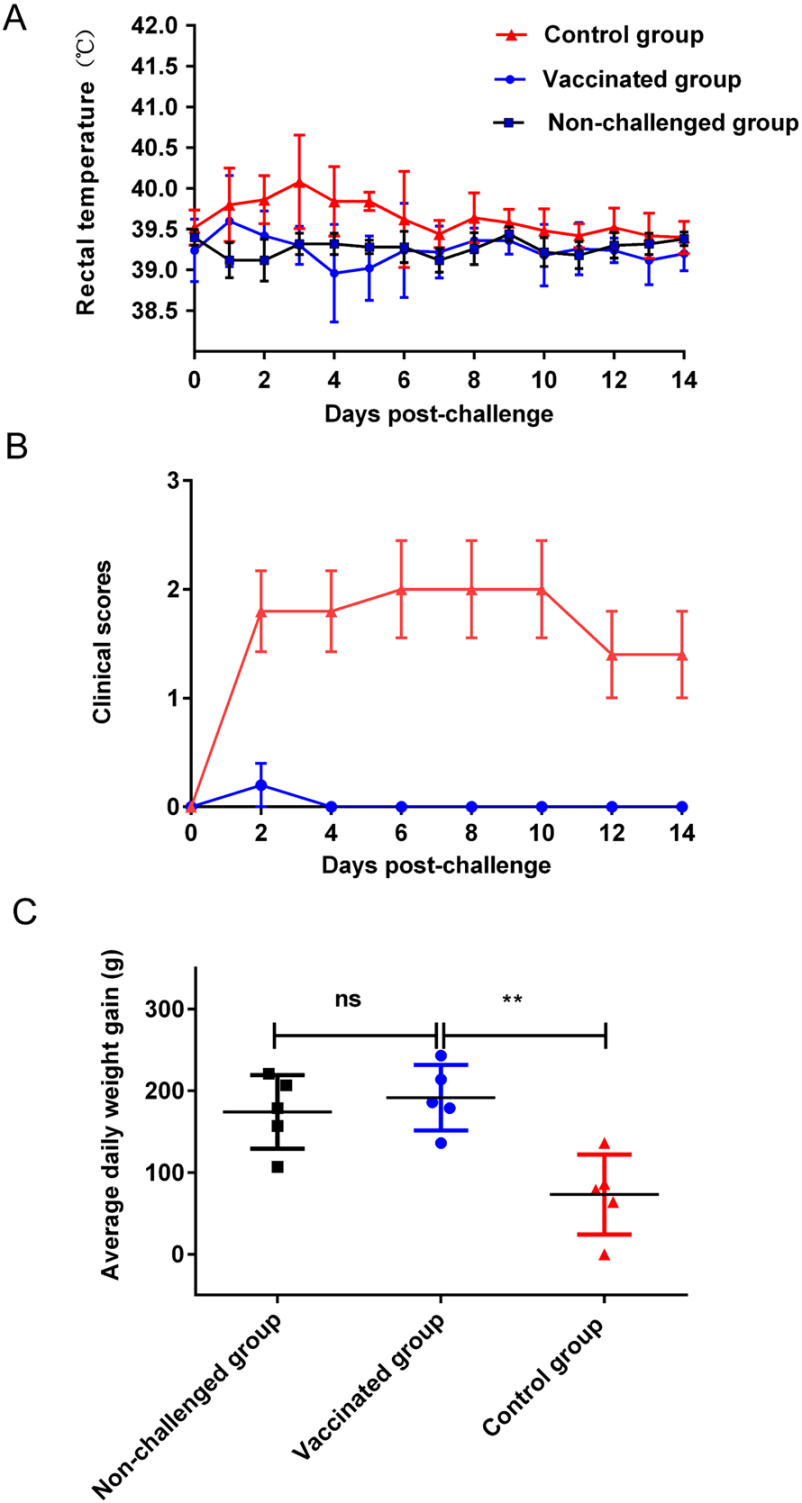
**Clinical scores and average weight gain of the sheep.** **A** Rectal temperature was monitored daily for 14 days after infection. **B** Clinical scores of vaccinated and control sheep post-challenge. **C** Comparison of the average weight gain of sheep. The average body weight gain significantly differed between the vaccinated group and the control group (*P* < 0.01).

Following a challenge with the OPIV3 TX01 F6 strain (4 mL/sheep at 1 × 10^7.5^ TCID_50_/mL), the total daily clinical symptoms were recorded, and the results showed that symptoms appeared 2–14 days after exposure to the virus in the control group. These symptoms were relatively obvious and could last for several days (Table 1). The sheep in the control group developed clinical signs of depression, cough, asthma, nasal discharge and anorexia post-challenge. It should also be noted that there were individual differences in clinical symptoms. These results are consistent with previous reports [30]. The vaccinated sheep were protected with fewer clinical signs than were the unvaccinated controls. After challenge, the body temperature of all sheep vaccinated with the inactivated OPIV3 vaccine prototype remained within the normal range until the end of the study. In contrast, the animals in the control group showed an increase in body temperature, reaching values higher than 40.0 °C for at least 1 day. During the 0 to 14 days of viral infection, 4 sheep had body temperatures greater than 40.2 °C twice (Figure 4A).

**Table 1:** **Clinical signs**

Group	Clinical signs: 1. Depression 2. Cough 3. Asthma 4. Nasal discharge^a^							
	0	2	4	6	8	10	12	14
Non-challenged	**–/–/–/–**^b^	**–/–/–/–**	**–/–/–/–**	**–/–/–/–**	**–/–/–/–**	**–/–/–/–**	**–/–/–/–**	**–/–/–/–**
	**–/–/–/–**	**–/–/–/–**	**–/–/–/–**	**–/–/–/–**	**–/–/–/–**	**–/–/–/–**	**–/–/–/–**	**–/–/–/–**
	**–/–/–/–**	**–/–/–/–**	**–/–/–/–**	**–/–/–/–**	**–/–/–/–**	**–/–/–/–**	**–/–/–/–**	**–/–/–/–**
	**–/–/–/–**	**–/–/–/–**	**–/–/–/–**	**–/–/–/–**	**–/–/–/–**	**–/–/–/–**	**–/–/–/–**	**–/–/–/–**
	**–/–/–/–**	**–/–/–/–**	**–/–/–/–**	**–/–/–/–**	**–/–/–/–**	**–/–/–/–**	**–/–/–/–**	**–/–/–/–**
Vaccinated	**–/–/–/–**	**–/–/–/–**	**–/–/–/–**	**–/–/–/–**	**–/–/–/–**	**–/–/–/–**	**–/–/–/–**	**–/–/–/–**
	**–/–/–/–**	**–/–/–/–**	**–/–/–/–**	**–/–/–/–**	**–/–/–/–**	**–/–/–/–**	**–/–/–/–**	**–/–/–/–**
	**–/–/–/–**	**–/–/–/–**	**–/–/–/–**	**–/–/–/–**	**–/–/–/–**	**–/–/–/–**	**–/–/–/–**	**–/–/–/–**
	**–/–/–/–**	**–/–/–/–**	**–/–/–/–**	**–/–/–/–**	**–/–/–/–**	**–/–/–/–**	**–/–/–/–**	**–/–/–/–**
	**–/–/–/–**	**–/ + /–/–**	**–/–/–/–**	**–/–/–/–**	**–/–/–/–**	**–/–/–/–**	**–/–/–/–**	**–/–/–/–**
Control	**–/–/–/–**	**+/–/+/–**	**+/–/+/–**	**+/+/+/–**	**+/+/+/–**	**+/+/+/–**	**+/+/+/–**	**+/+/+/–**
	**–/–/–/–**	**+/+/+/–**	**+/+/+/–**	**+/+/+/–**	**+/+/+/–**	**+/+/+/–**	**+/–/–/–**	**+/–/–/–**
	**–/–/–/–**	**+/–/–/–**	**+/–/–/–**	**+/–/+/–**	**+/–/+/–**	**+/–/+/–**	**+/–/–/–**	**+/–/–/–**
	**–/–/–/–**	**+/–/–/–**	**+/–/–/–**	**+/–/–/–**	**+/–/–/–**	**+/–/–/–**	**+/–/–/–**	**+/–/–/–**
	**–/–/–/–**	**+/–/–/+**	**+/–/–/+**	**+/–/–/–**	**+/–/–/–**	**+/–/–/–**	**+/–/–/–**	**+/–/–/–**

^a^Clinical signs (depression/cough/asthma/nasal discharge) were recorded separately from day 0 to day 14, and use “+” indicated that one of the above clinical symptoms occurred. “-” indicated that no above clinical symptoms occurred

^b^ “–/–/–/–” indicated that none clinical symptoms occurred

The clinical signs typical of PIV3 infection were examined, and the overall clinical reaction score was calculated. However, none of the four vaccinated animals experienced cough, asthma, or nasal discharge after the challenge. One of the sheep appeared depressed at 2 days post-challenge (dpc). A graphical representation of the daily clinical score for each group is shown in Figure 4B. In addition, the average body weight gain was significantly different between the vaccinated and control groups (*P* < 0.01) (Figure 4C).

**Figure 5:**
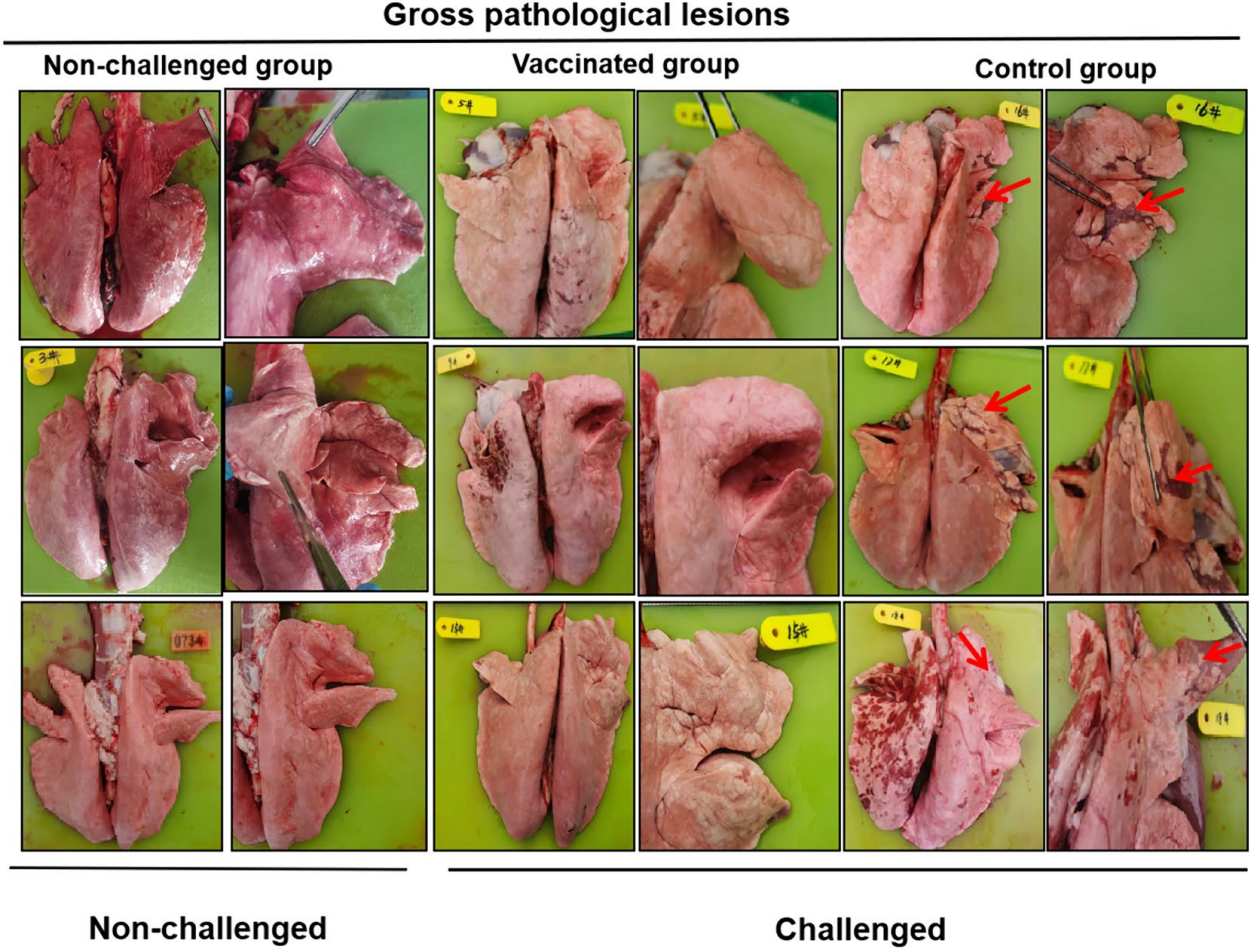
**Gross pathological lesions in sheep lungs.** Multifocal red areas (red arrows) were observed in the lungs of unvaccinated sheep but not in the lungs of vaccinated sheep or those of the nonchallenged group. The lesions were mainly concentrated in the right lung lobule. Lung consolidation was mild to moderate.

The sheep in the control group exhibited mild to moderate diffuse purple consolidation in the pulmonary lobules and interstitial lung disease. The lesions were mainly concentrated in the right upper lobe. The sheep lungs in the vaccinated group exhibited normal gross pathology (Figure 5). No pathological changes in the heart, liver, spleen or kidney were observed in the other groups (data not shown).

**Figure 6:**
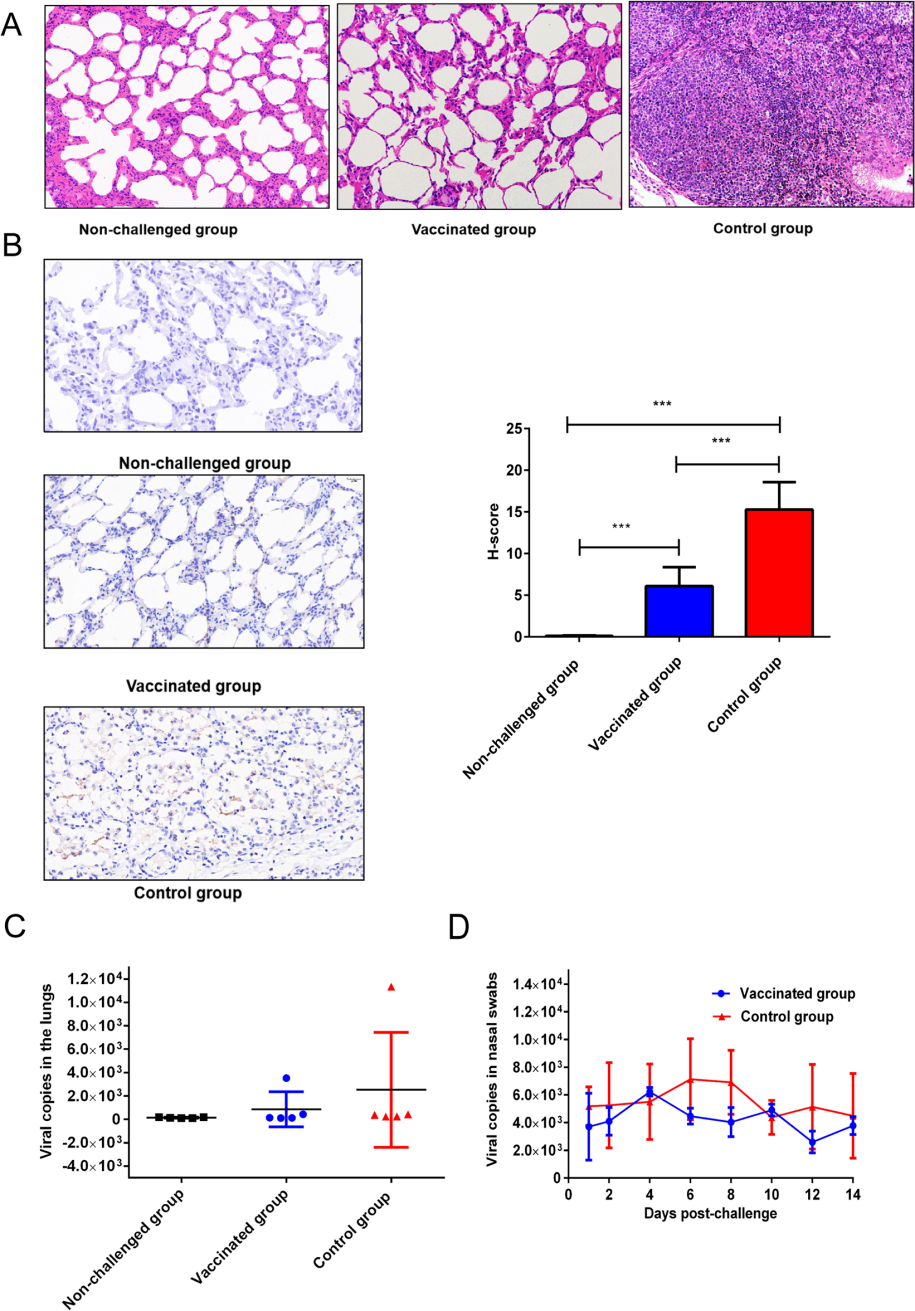
**Histopathological examinations, viral loading and viral shedding. A** Histopathological examination of sheep lungs at 14 dpc. The lungs of the sheep in the vaccinated group were necropsied at 14 dpc (magnification, × 200). Lung sections from control sheep showed severely thickened alveolar septa, expansions of the alveolar interstitium, congestion, macrophage infiltration, and compensatory emphysema. The results revealed the appearance of viral pneumonia with interstitial lymphocytic infiltrates (magnification, × 200). **B** Formalin-fixed lung sections were subjected to OPIV3 load evaluation by IHC. **C** The viral load was evaluated by RT-qPCR. **D** Viral shedding was evaluated by RT-qPCR.

Histopathological analysis revealed that the alveolar walls were obviously thickened, the blood vessels were dilated and congested, and there was fibrous histiocytosis and severe immune cell infiltration. No lesions were observed in the control group (Figure 6A). There were no obvious histological abnormalities in other organs or tissues (data not shown).

N protein expression in the lungs of OPIV3-infected animals was determined by immunohistochemistry in association with viral load and clinicopathological features. The mean N protein level in the control and vaccinated groups was greater than that in the nonchallenged group (15.278, 6.102 vs. 0.124; *P* < 0.001). The data are shown as one-way ANOVA data for the vaccinated group vs. the nonchallenged group (6.102 vs. 0.124, *P* < 0.001) and for the control group vs. the vaccinated group (15.278 vs. 6.102, *P* < 0.001). Overall, the results showed different staining intensities for the OPIV3 N protein in each group because of differences in viral load. There was obviously lower expression of OPIV3-N in the lungs of the vaccinated group than in the lungs of the control group. (Figure 6B).

For further analysis of viral load and shedding, RT‒qPCR was used to evaluate the protection afforded by the OPIV3 vaccine candidates. The viral loads in the lungs of the vaccinated group were 860.6 ± 669.4 copies/µL (*n* = 5), whereas those in the control group were 2520 ± 2202 copies/µL (*n* = 5). The number of viral copies/µL in the lungs of the vaccinated group was lower than that in the lungs of the control group. The lungs of three of the sheep in the vaccinated group were negative for OPIV3 according to RT‒qPCR (CT ≥ 33). The peak viral loads were observed in the control group and vaccinated group, in which there were 11.327 × 10^3^ copies/µL and 3.527 × 10^3^ copies/µL, respectively. Individual differences are shown in Figure 6C. Moreover, the liver, heart, kidney and spleen of the challenge sheep were negative for OPIV3 (data not shown). The sheep were positive for OPIV3 nasal shedding post-challenge. The peak nasal shedding occurred on Day 4 post-challenge in the vaccinated group and on Day 6 post-challenge in the control group. Compared with those in the vaccine groups, the viral genome loads in the nasal swabs of the control group were greater on Days 1, 2, 6, 8, 12, and 14 (Figure 6D). The data are shown as the mean ± SEM.

### Virus neutralization antibody response

**Figure 7:**
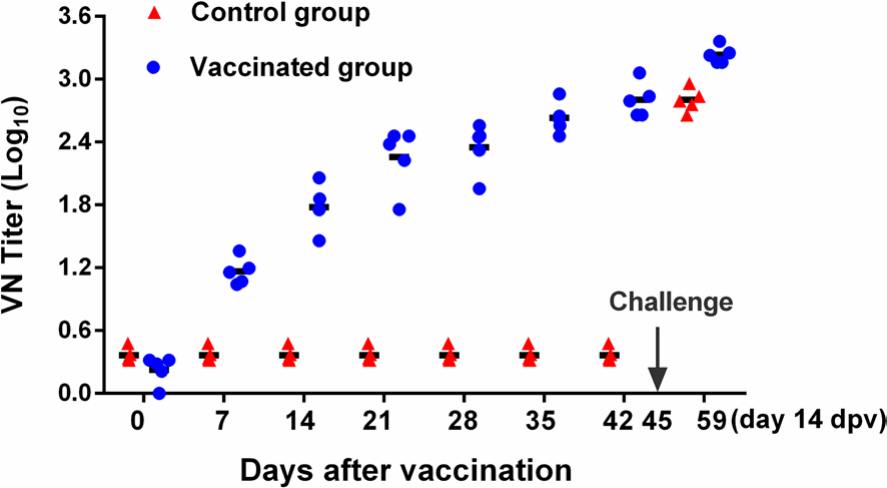
**Virus neutralization antibody response.** Groups of sheep (*n* = 5) were vaccinated or not vaccinated at 0 and 21 days and challenged with OPIV3 at 45 dpv. Scatter plot showing the neutralizing antibody titres in the vaccinated group (blue circle) and control group (red triangle). The symbols represent individual animals, and the short lines represent the mean VN titres for each group. The data are shown as the mean ± S.D.

The induction of virus-neutralizing (VN) antibodies after vaccination can be a key protective immune response. Thus, we evaluated the levels of VN antibodies. In the vaccinated groups, VN antibodies were detected at 7 dpi. After 14 days of vaccination, the VN antibody titre in sheep was 1.777 ± 0.217 log_10_ (*n* = 5), whereas that in the control group was 0.364 ± 0.067 log_10_ (*n* = 5). The VN antibody titre continuously increased post-vaccination (dpv) and with a booster post-challenge in vaccinated sheep. After 21 days and 42 days of vaccination, the neutralizing antibody titres in sheep were 2.256 ± 0.295 log_10_ (*n* = 5) and 2.802 ± 0.165 log_10_ (*n* = 5), respectively. In contrast, the neutralizing antibody titres in the control group increased only after the challenge (Figure 7). The data are shown as the mean ± S.D.

## Discussion

Vaccination is among the most effective ways to prevent respiratory diseases caused by parainfluenza virus type 3 diseases [31, 32]. Compared to live vaccines, inactivated vaccines have attracted much attention due to their safer profile and easier transport and storage [33]. Inactivated viruses have been traditionally used for vaccine development, and such vaccines are safe and effective for the prevention of viral diseases such as coronavirus disease 2019 (COVID-19), influenza, foot and mouth disease, and polio [34–36].

In our previous research, the PIV3 strain was isolated from sheep suffering severe respiratory disease in China. We have previously described the phylogenetic analysis and pathogenicity of PIV3 in sheep [30]. By analysing the important parameters relevant to the evaluation of treatment efficacy (body temperature/clinical signs/gross pathological/viral shedding and viral loading/VN antibody titres), significant differences in clinical signs and weight changes were found between the vaccinated sheep and the controls after viral challenge. Compared with BPIV3 and HPIV3 vaccine studies, there are fewer PIV3 vaccine studies in sheep and goats, and these studies mainly date from the 1970s and 1980s [37–40]. Our results show that the OPIV3 vaccine tested in the current study induced higher levels of antibodies than those previously reported, and the percentage of gross lung lesions decreased significantly [37, 39]. These differences may be due to differences in the candidate vaccine strains and/or the different adjuvants used [42]. The complete gene sequences of the TX01 strain were uploaded to GenBank in 2021 [30]. ISA 206 (Seppic, France) adjuvanted vaccines stimulate protective immune responses in pigs, cattle, and sheep within a short period, with little or no toxicity or local reactivity [41]. ISA 206, the mineral-based oil described earlier, readily forms a water-in-oil-in-water emulsion [42].

Although the breeds and ages of the sheep used in the experiment differed from those used in the previous study, the vaccine strains and the immunization and challenge doses were also different, and the inactivated parainfluenza vaccine reduced the severity of respiratory diseases caused by parainfluenza, which is consistent with previous studies.

The main clinical signs produced by the PIV3 challenge in our studies are consistent with clinical symptoms observed in other PI3V challenge experiments [30, 43]. It is important to point out the reasons for the differences in daily weight gain of sheep may be due to season, environment, breed, and age. The OPIV3 TX01 wild-type strain was isolated from the lungs of both groups after gross dissection (data not shown). On the one hand, the pathogenic capacity of OPIV3 in the lungs was shown; on the other hand, the challenge dose of OPIV3 requires further study as the natural infection volume is likely to be lower than the challenge dose, and the infection mode also differs from the challenge mode. In addition, the breed of sheep and individual differences should be considered. Studies on vaccine immune dose, minimum dose, and treatment duration will be performed in the future to establish the protective antigen payload in the vaccine and provide insights into the evaluation of vaccine immunogenicity.

Previously, researchers have shown that vaccination against the viral component in virus-enhanced bacterial infections of the respiratory tract prevents a predisposition to bacterial superinfection [44].

The lambs vaccinated against PIV3 virus were successfully protected against the clinical illness associated with the virus and were also protected in part against the effects of bacterial superinfection. In addition, both inactivated vaccines and attenuated intranasal PIV3 vaccines can reduce the severity of pneumonia lesions and lamb mortality associated with secondary *Pasteurella haemolytica* infection [31, 38, 44]. Consequently, once PIV3 vaccination is widely used, the possibility of secondary bacterial infection will be reduced. Our future studies will include in-depth research on the prevention and control strategies of PIV3-induced respiratory diseases.

In conclusion, this study provides encouraging data regarding the safety and efficacy of the PIV3 vaccine for sheep and offers new ideas for the prevention and control of respiratory diseases.

## Data Availability

The data that support the findings of this study are available from the corresponding author upon reasonable request. All the data generated or analysed during this study are included in this published article.
